# Target Product Profile for a Diagnostic Assay to Differentiate between Bacterial and Non-Bacterial Infections and Reduce Antimicrobial Overuse in Resource-Limited Settings: An Expert Consensus

**DOI:** 10.1371/journal.pone.0161721

**Published:** 2016-08-25

**Authors:** Sabine Dittrich, Birkneh Tilahun Tadesse, Francis Moussy, Arlene Chua, Anna Zorzet, Thomas Tängdén, David L. Dolinger, Anne-Laure Page, John A. Crump, Valerie D’Acremont, Quique Bassat, Yoel Lubell, Paul N. Newton, Norbert Heinrich, Timothy J. Rodwell, Iveth J. González

**Affiliations:** 1 Foundation for Innovative New Diagnostics (FIND), 9 Chemin des Mines, 1202 Geneva, Switzerland; 2 Special Programme for Research & Training in Tropical Diseases (TDR), World Health Organization, Avenue Appia 20, 1211, Geneva 27, Switzerland; 3 Hawassa University, College of Medicine and Health Sciences, Department of Pediatrics, Hawassa, Ethiopia; 4 World Health Organization, 20 Avenue Appia, 1211, Geneva 27, Switzerland; 5 Institute of Infectious Diseases and Epidemiology, Tan Tock Seng Hospital, Singapore, Singapore; 6 ReAct Europe, Uppsala University, Box 256, 751 05, Uppsala, Sweden; 7 Epidemiology and Population Health, Epicentre, Paris, France; 8 Centre for International Health, University of Otago, PO Box 56, Dunedin, 9054, New Zealand; 9 Duke Global Health Institute, Box 90519, Duke University, Durham, NC, 27708, United States of America; 10 Division of Infectious Diseases and International Health, Box 102359, Duke University Medical Center, Durham, NC, 27710, United States of America; 11 Swiss Tropical and Public Health Institute, 4002 Basel, Switzerland; 12 Department of Ambulatory Care and Community Medicine, University of Lausanne, Bugnon 44, 1011, Lausanne, Switzerland; 13 ISGlobal, Barcelona Ctr. Int. Health Res. (CRESIB), Hospital Clínic—Universitat de Barcelona, Barcelona, Spain; 14 Centro de Investigação em Saúde de Manhiça (CISM), Maputo, Mozambique; 15 Centre for Tropical Medicine and Global Health, University of Oxford, Oxford, United Kingdom; 16 Mahidol-Oxford Tropical Medicine Research Unit, Faculty of Tropical Medicine, Mahidol University, Bangkok, Thailand; 17 Lao-Oxford-Mahosot Hospital-Wellcome Trust Research Unit, Vientiane, Lao PDR; 18 Division for Infectious Diseases and Tropical Medicine, Medical Center of the University of Munich (LMU), Munich, Germany; German Center for Infection Research, Munich partner site, Leopoldstr 5, D-80802, Munich, Germany; McGill University Health Centre, CANADA

## Abstract

Acute fever is one of the most common presenting symptoms globally. In order to reduce the empiric use of antimicrobial drugs and improve outcomes, it is essential to improve diagnostic capabilities. In the absence of microbiology facilities in low-income settings, an assay to distinguish bacterial from non-bacterial causes would be a critical first step. To ensure that patient and market needs are met, the requirements of such a test should be specified in a target product profile (TPP). To identify minimal/optimal characteristics for a bacterial vs. non-bacterial fever test, experts from academia and international organizations with expertise in infectious diseases, diagnostic test development, laboratory medicine, global health, and health economics were convened. Proposed TPPs were reviewed by this working group, and consensus characteristics were defined. The working group defined non-severely ill, non-malaria infected children as the target population for the desired assay. To provide access to the most patients, the test should be deployable to community health centers and informal health settings, and staff should require <2 days of training to perform the assay. Further, given that the aim is to reduce inappropriate antimicrobial use as well as to deliver appropriate treatment for patients with bacterial infections, the group agreed on minimal diagnostic performance requirements of >90% and >80% for sensitivity and specificity, respectively. Other key characteristics, to account for the challenging environment at which the test is targeted, included: i) time-to-result <10 min (but maximally <2 hrs); ii) storage conditions at 0–40°C, ≤90% non-condensing humidity with a minimal shelf life of 12 months; iii) operational conditions of 5–40°C, ≤90% non-condensing humidity; and iv) minimal sample collection needs (50–100μL, capillary blood). This expert approach to define assay requirements for a bacterial vs. non-bacterial assay should guide product development, and enable targeted and timely efforts by industry partners and academic institutions.

## Background

Acute fever is one of the most common presenting symptoms in healthcare facilities in resource limited countries, and acute febrile illness (AFI) results in considerable morbidity and mortality annually [1–3]. Until recently, malaria was considered to be the predominant cause of fever in many endemic regions of the world. However, with the increased use of malaria rapid diagnostic tests (RDTs), it has become apparent that a much smaller proportion of fevers are in fact caused by malaria parasites than previously understood [4]. Recent global studies of fever etiology have highlighted a great diversity of causative pathogens that vary depending study population, with considerable variability in prevalence and diversity both within and between countries [5–11].

The decreased incidence of malaria, a paucity of even rudimentary microbiology facilities due to a lack of human and financial resources, missing rapid diagnostic assays and the absence of reliable clinical signs and symptoms has led to an increased uncertainty of algorithm-driven empiric use of antimicrobials [11]. The overuse of antimicrobial drugs contributes to the global increase of antimicrobial resistance (AMR) and it is estimated that currently 500,000 people die annually from infections caused by drug resistant pathogens [12,13]. In low and middle income countries (LMICs), antimicrobial overuse is probably greater, given the paucity of diagnostic tests, fewer controls on antimicrobial dispensing, and a lack of antimicrobial stewardship programs [2,11,14] which likely contributes to an antibiotic resistance problem of alarming magnitude [15,16]. The importance of diagnostic tests for evidence-based treatment has been recognized by the World Health Organization (WHO) and other global health stakeholders as a direct means of reducing the inappropriate use of antimicrobials and decreasing selective pressure on microbes toward drug resistance [17]. Further an executive order on AMR published by the president of the United States highlights the need for multidisciplinary approaches including the development of point of care diagnostic [18].

In addition to limiting inappropriate antibiotic use, targeted and evidence-based treatment will improve patient outcomes, reduce adverse events, and provide economic benefit for the healthcare system and the patient [19]. Excessive health care spending is of particular concern in many LMICs, where health care spending is primarily out-of-pocket expenditure [20]. A number of studies have highlighted the lack of available assays to fill this diagnostic gap in resource-limited settings [13,18–20]. Assays for detecting host biomarkers associated with bacterial fevers, such as C-reactive protein (CRP) and procalcitonin (PCT) are used in hospitals in Europe to differentiate between bacterial and non-bacterial infections [21], and guide triage and treatment decisions [22–24]. However, malaria, co-infections such as HIV, and the presence of poverty-related diseases and conditions such as malnutrition have been shown to confound the interpretation of CRP and PCT results, limiting the value of these assays in LMICs [25–29]. Thus, while the use of existing CRP or PCT tests could, in some settings, cost-effectively improve the management of fever when compared with current practice [2,30], there is likely to be considerable further advantage in improved biomarker tests that will be applicable to a wider range of patients, once available. In recent years a number of promising assays have been described in the literature that use single or combinations of biomarkers to predict bacterial versus non-bacterial causes of fever. These assays, recently described in a systematic review, might represent encouraging alternatives to CRP or PCT assays [31].

As novel platforms and technologies for biomarker detection and pathogen-specific testing are being developed in the near term (5-years), it is important to ensure they meet the needs of end-users. In particular, it is essential that the technological challenges faced at lower levels of the health care system in resource-limited settings are taken into consideration during the product development process [32].

Here, we describe the TPP definition process, involving a consortium of global health and diagnostic experts, and the final consensus characteristics for a test to distinguish bacterial from non-bacterial infections. The core group included individuals from the World Health Organization (WHO), ReAct–Action on Antibiotic Resistance, Médecins sans Frontières (MSF) Access Campaign and the Foundation for Innovative New Diagnostics (FIND). These organizations convened a fever biomarker diagnostics meeting in September 2015 [33], and established a TPP working group to develop the product specifications described here.

## Methodology

Following a review of the current biomarker landscape [31] the need for TPPs was identified. Preparing the presented TPPs for an assay to differentiate between bacterial and non-bacterial infections was a collective effort initiated prior to the biomarker meeting in September 2015 (Geneva, Switzerland) [33]. An adapted Delphi approach with three rounds of input was used to obtain an expert driven consensus for TPPs. In preparation for the meeting a first draft of TPP characteristics was shared among the convening organizations (S1 File). Assay and platform characteristics outlined in the TPPs had previously been agreed upon by experts and as the listed characteristics were considered of being of the highest importance to guide product development in compliance with ISO13485 and 21CFR Part 820. The listed characteristics are therefore used in all FIND TPPs (http://www.finddx.org/target-product-profiles/). TPP characteristics are described as either “acceptable” or “desired.” An assay’s attributes or characteristics are defined as “acceptable” when they are considered minimum criteria and thresholds that must be reached in order for the diagnostic tool to be useful to health care providers and beneficial for patients in the defined site of use. “Desired” characteristics refer to optimal attributes or characteristics that are highly desirable by healthcare workers and patients.

The first draft was based on a review of relevant literature related to the selected characteristics (S2 File). Prior and during the biomarker meeting, recognized leaders in the field were identified for the final TPP working group (n = 15) based on their proven expertise in the field of non-malarial fever and/or biomarker studies in resource limited settings. The group was composed of experts in infectious disease, laboratory medicine, microbiology, global health, health economists, and diagnostic test development. To reduce the bias introduced by the expert driven approach, stakeholders working in a wide range of geographical regions and backgrounds were chosen. Based on the feedback in the first round a second TPP draft was prepared and circulated among the participants of the working group with the request for critical input (S3 File). Feedback and discussion points obtained in this second round of review were compiled in advance of a face-to-face consensus meeting (S4 File). Based on the feedback, attributes and characteristics were revised by FIND and prioritized for further discussion based on the level of concordance/discordance in the responses (Table 1). The draft characteristics were then presented by FIND to the meeting participants and discussed during a workshop held in Geneva in February 2016 which was attended in person or via conference link by 13 participants. If personal attendance was not possible the final TPP as well as discussion points were shared with active members (n = 14) of the initial TPP working group. All dissent and discussion points raised during the meeting were noted, and a concerted effort was made to reach full agreement. Final consensus TPPs were established after unanimous agreement by the group.

**Table 1:** Target product profile characteristics and their priority, expert consensus process 2015–16.

Priority for discussion	Characteristics
High	Target population, Level of health system, Analytical sensitivity/Limit of Detection (LOD), Diagnostic sensitivity/specificity, Target price, Time-to-result, Hands-on-time, Training requirements
Medium	Multiplexing, Ease of test performance, Sample type, Additional sample preparation, Throughput, Result stability, Shipping conditions, Equipment, Calibration, Connectivity, Reproducibility
Low	Target user, Volume, Reagent preparation, Waste disposal, Power supply, External maintenance, Data interpretation
Very low	Sample collection, Kit configuration, Control material, Analysis type, Biosafety, Storage conditions, Operation conditions, Water supply

A total of 35 test characteristics were included in the TPP, as shown in the list below. The following definitions were used for ranking. High: Large divergence between expert opinions, and/or significant implications for the final assay, and/or limited published evidence to guide decision. Medium: Some divergence between expert opinions, and/or Limited published evidence to guide decision, and/or review of wording after changes, and/or common characteristics modified after initial review. Low: Some divergence between expert opinions, and/or limited implications for the final assay, and/or strong published evidence available to guide decision. Very low: No divergence between expert opinion, and/or FDA/industry consensus already available.

## Results

### Scope of the test

The working group identified an assay to distinguish bacterial from non-bacterial infections as a top priority to improve fever management in low-resource settings [33]. The aim of such a test would be to rule in or rule out bacterial infections and therefore provide actionable, evidence-based treatment guidance. The acceptable population such a test should be focused on non-malarial acute pediatric febrile illness patients (2month– 12years) without severe disease (Table 2), as defined by WHO guidelines like the IMCI [34]. This was identified as the priority age group in order to reduce undifferentiated use of antimicrobials given the large incidence of febrile episodes in this population and the higher morbidity and mortality in children compared to older children and adults [35]. Ideally (“desired”) however, the target age range of a new assay should include all ages, including neonates. While in the “acceptable” the population is restricted to non-malarial patients the desired assay could also be performed without the inclusion of a downstream malaria test on the total febrile population. Primary health care facilities (level 1) and informal health care settings (level 0) with untrained or minimally trained staff represent the most diagnostically underserved facilities in many health care systems, particularly in resource-limited settings and were therefore identified as the target setting for this assay (Table 2). Based on a 2006 review by Lim *et al*., an assay implemented at these levels will impact the largest number of febrile patients [36]. The TPP Working Group agreed that a test with high sensitivity is important to improve treatment outcomes through early detection of patients in need of treatment.

**Table 2:** Acceptable and desired target product profile characteristics focused on the scope of the test, as defined by an expert consensus process 2015–16.

Characteristic	Acceptable ("must have")	Desired ("would like")	Reference
**Goal**	Rapid, biomarker-based testing to differentiate between bacterial and non-bacterial infections to guide antimicrobial treatment.^a^		Expert consensus
**Target population**	Children with non-severe, non-malarial acute fever presenting at health facilities. ^b^^,^^c^^,^^d^	Total febrile population (including neonates) presenting with fever. ^b^^,^^c^^,^^d^	[4,35]
**Target level of health system**	Level 1, passive case finding	Level 0	[37]
**Target user**	Healthcare worker	Trained lay person	[38]
**Price of individual test (Ex works)**	5 USD ^e^	<1 USD ^e^	Expert consensus
**Analytical sensitivity/Limit of detection**	Limit of detection should be such that it allows clinically relevant performance as defined below		Expert consensus
**Diagnostic sensitivity to differentiate between bacterial and non-bacterial infections**	≥90%	≥95%	Expert consensus
**Diagnostic specificity to differentiate between bacterial and non-bacterial infections**	≥80%	≥90%	Expert consensus

^a^ Biomarker: nucleic acid, proteins or any other analytes that is found to be able to differentiate between bacterial and non-bacterial infections

^b^ Non-severe: definition according to IMCI guidelines[34]

^c^ Acute fever: less than 14 days

^d^ Fever: >37.5°C at presentation or within last 48h

^e^ Ex works as defined by Incoterms 2010 standards

Further to reduce antibiotic overuse confidence in test ability to detect bacterial infection is needed (high sensitivity). This needs to be combined with decent specificity to limit false positive result. The working group agreed that a specificity of ≥80% would be a strong improvement on the current situation, where empiric antibiotic use is the norm. A recent study from South East Asia demonstrated that 38% of all febrile patients received an antimicrobial and that this was not focused on bacterial infections alone [2]. In Africa, the overuse of antibiotics seems to be even larger, with between 50% and 80% of febrile outpatients receiving antibiotics [39].Test price estimates are based on expert opinion, and estimates of what is acceptable (<5 USD) or desirable (<1 USD) out-of-pocket costs in LMICs. As with other RDTs (e.g., for HIV or malaria), it is expected that prices would decline over time as market demand increases, costs of production reduce due to volume-based efficiencies of scale, and competition increases due to multiple suppliers [40].

### Test and operational characteristics

The group agreed that, in the absence of pathogen-specific tests, at a minimum a new fever assay needs to be able to distinguish between bacterial and non-bacterial infections, after malaria has been ruled out. However, an additional strength would be the inclusion of additional analytes, particularly a pathogen-specific marker for the detection of malaria. In some settings, other geographically relevant pathogens (e.g., Dengue, *O*. *tsutsugamushi* [2]) as well as markers of disease severity would be “desired” features (Table 3).

**Table 3:** Acceptable and desired target product profile characteristics focused on operational characteristics, as defined by an expert consensus process 2015–16.

Characteristic	Acceptable ("must have")		Desired ("would like")	Reference
**Multiplexing**	≥1 analyte		≥1 analyte plus pathogen specific testing (priority: malaria)	Expert consensus
**Ease of test performance**	≤2 timed steps during assay performance		No timed step during the assay performance	Expert consensus
**Sample type**	Capillary blood or urine		Capillary blood or any less invasive sample than blood, like: Saliva	Expert consensus
**Volume**	- Capillary blood: - patients > 6 years[41] - 100μL (~ 4 drops); - pediatric patients 2–6 years[41]– 50–100μL (~2 drops); - Saliva: < 0.5mL - Urine: ~1mL of urine		- Capillary blood: 25μL (~1 drop) for all age groups - Saliva: <0.5mL for saliva	Expert consensus
**Sample collection**	Transfer and quantification device included in the test			Industry standard
**Additional sample preparation**	1 sample-processing steps		None required	Expert consensus
**Kit configuration**	No additional reagents outside of the kit required (including gloves)			Industry standard
**Process control**	Internal control to provide test validity and acceptance			Expert consensus
**Batch/Quality control**	Positive and negative controls required to monitor the quality of kit			Expert consensus
**Reagent preparation**	- Minimal of one additional step to prepare prior to use - No (precise) measuring required.		No additional reagents required, everything is provided ready-to-use	[32]
**Time to result (per sample)**	<2 hours		<10min	Expert consensus, Ivanova *et*. *al*. in preparation
**Hands on time**	Total hands-on-time should be <5 min		Total hands on time should be <1 min	Expert consensus
**Sample throughput**	Ability to test individual samples or multiple samples if needed (no need for batching)			Expert consensus
**Result stability**	≥15 min		≥1 hour	Industry standard
**Assay type**	Qualitative		Quantitative	Expert consensus
**Biosafety**	No need for a biosafety cabinet; basic safety procedures need to be followed			[42]
**Waste disposal**	Biohazard waste	- Testing device - Compostable plastics for minimal environmental impact		[42]
**Storage conditions and Self-life**	- 12 month at fluctuating temperature (0–40°C) - ≤90% relative humidity - No controlled temperature required	- 24 month at fluctuating temperature (0–50°C) - ≤90% relative humidity - No controlled temperature required		Expert consensus
**Operation conditions**	- Between 5°C—40°C - ≤90% humidity	- Between 5°C—45°C - ≤90% humidity		Expert consensus
**Shipping conditions**	Shipping without cold chain; should tolerate stress during transport (≤72h at +50°C)			Expert consensus
**Training requirements**^**a**^	<2 days including proficiency panel	<0.5 day including proficiency panel		[32]
**Equipment (instrument external to test)**	Small, robust, dust-resistant, portable or hand-held integrated instrument that must operate on battery	Reusable instrumentation not required		Expert consensus
**Power supply**	Battery or solar powered	None required		[32]
**Water supply**	No external water required			Industry standard
**External maintenance**	- Preventative maintenance at 2 year or >4000 samples; simple with only minimal expertise - Maintenance alert should be included.	None required		Industry standard
**Calibration**	Remote calibration or auto-calibration	No calibration required		Industry standard
**Data output**	Qualitative	Non-ambiguous results displayed (e.g. bacterial/non-bacterial–yes or no)		Expert consensus
**Data interpretation**^b^	Minimal interpretation required	No interpretation required		Expert consensus
**Connectivity**	Not required	Wireless connectivity		Isaak *et*. *al*. in preparation
**Reproducibility**	>95% standard deviation between repeats			[43]

^a^ Training to use the test, not including clinical/treatment implications and consequences.

^b^ Interpretation of the test result, not the clinical/treatment conseque

Capillary blood, saliva and urine were considered “acceptable” sample matrices (Table 3). Experts note that while for adult patients urine is an accessible and ideal non-invasive clinical sample, in small children obtaining urine can be difficult and time-intensive; with one study reporting a median of 25 minutes to obtain urine from children using either pads, bags, or clean catch methods [44]. Thus, urine does not represent an ideal sample for point-of-care testing in our target setting, given the importance of a test appropriate to children. Similarly, obtaining venous blood from infants and small children can be challenging; the TPP Working Group agreed that blood volumes should be restricted to ~50–100μL (~2 drops) or ~100 μL (~4 drops) for children or adults, respectively (Table 3).

Time-to-result is defined as the time between sample collection and reporting the result. It was agreed that the ideal test should be used within the time constraint of a patient consultation, while the maximum “acceptable” time to result would be two hours (Table 3). The latter estimate is based on clinical experience with the amount of time patients are willing to wait, particularly in rural health centers with large catchment areas that require patients to travel long distances.

Depending on the catchment area, season, and geographical location, the number of patients presenting at health facilities can fluctuate and health providers need to be able to perform one or many tests simultaneously, comparable to the current malaria RDTs. Hence, a hands-on time of <5 minutes was defined as acceptable and under one minute as desirable. The TPP Working Group agreed that, based on the target setting and user of this test, training requirements should be minimal, and enable proficiency of the end user (with limited biomedical training) after a maximum of two training days (Table 3). Further, the group also identified connectivity as a “desired” characteristic. This is particularly the case as new barcoding and connectivity technologies allow for improved records including data on test type, lot numbers, manufacturing dates and expiry dates. Connectivity options can also allow for closer integration of the test with electronic patient management algorithms that have been shown to improve antibiotic targeting [45].

## Discussion

Establishing and disseminating the TPPs of diagnostic tests identified as high priority will help guide product development teams. In the case of AFIs, this would be expected to accelerate the development of much needed tools. In addition, by making the approach and analysis transparent, and distinguishing areas of broad expert agreement as well as divergence, groups involved in diagnostic test development can make informed decisions about product features and performance specifications.

Accurate diagnosis of febrile illness is critical for appropriate, individualized patient care and improved public health, as “without diagnostics, medicine is blind” [46]. Unfortunately, the wide range of causative agents of fever, and in many cases non-specific clinical features of diverse infections make pathogen-specific diagnoses challenging. While the challenges associated with identifying causative agents of fever are universal, the lack of human, financial and structural capacity significantly complicates patient care in LMICs. This is particularly true for level 1 and level 0 where, as described by Lim and colleagues, the largest proportion of patients in Africa, Asia, and Latin America access care [36]. Therefore, diagnostic tests at these levels need to fulfill the WHO proposed Affordable Sensitive Specific User-friendly Rapid and Equipment-Delivered (ASSURED) criteria [38,47].

As a first step to address the need to provide adequate treatment to many millions of patients, the TPP presented here has been established to guide the development of new diagnostic assays, or the adaptation of available assays. The diagnostic characteristics described can be viewed as guides to product development; the final performance characteristics, including clinical sensitivity and specificity, would need refinement based on population needs and market dynamics. However, while the current group slightly favored sensitivity over specificity in this performance trade-off, the defined diagnostic parameters (sensitivity/specificity) are in line with previous estimates for acute lower respiratory infection (ALRI) [36]. The 2006 study by Lim and colleagues concluded that a similar test, targeted only at ALRI, with 95% sensitivity and 85% specificity would save ~ 405,000 children annually [36]. An additional challenge is the lack of reference standards and curated specimen banks for bacterial and non-bacterial infections against which biomarker tests can be evaluated. The desired use of a multiplexed or multi-analyte approach could further help to conserve human and economic resources. In most instances, however, given its prevalence, global burden of disease, public health importance, and curative treatment regimens, a biomarker plus malaria combi-test should be prioritized. Market analyses remain to be conducted to understand what the sustained demand for such tests would be, based on geography and health system. Defining a target price for the assay is also a complex challenge as the market for such an assay is not well defined in LMICs. For the purposes of this TPP, we assumed that price pressures will be similar to those observed in the current RDT market for malaria [40].

Although product developers should aim to meet the defined characteristics as closely as possible, given the paucity of available assays, some flexibility in the specifications is appropriate. Some of the “acceptable” criteria were chosen deliberately general and matching those might result in reduced uptake of an assay. We acknowledge that depending on the technical approach taken by developers, the small sample volumes proposed by us can pose a challenge for pathogen-specific tests with low magnitude bacteremia or antigenemia [48]. Another limitation of the presented TPP is the extent to which it had to rely on expert opinion as specific studies around health seeking behavior were not available due to the lack of published targeted investigations. This lack of information identified by our group should encourage targeted investigations into the health care seeking behavior as well as socio-economic barriers associated with fever diagnostic. The inclusion of stakeholders from diverse backgrounds, with a wide range of experiences in different countries and settings, aimed to counteract the bias potentially introduced by this expert-based approach. A further limitation might be the exclusion of industry stakeholders a decision taken to allow the development of TPPs that exclusively focused on patient health care provider’s needs.

The availability of fever biomarker assays, even with excellent performance characteristics, is unlikely to have sustained impact unless endorsed and incorporated into corresponding WHO treatment guidelines, similar to the *P*. *falciparum* rapid tests [34]. For example, integrating a biomarker test within the Integrated Management of Childhood Illness (IMCI; WHO) or Integrated Management of Integrated Management of Adolescent and Adult Illness (IMAI; WHO) [34,49] would add more classifications to the existing decision algorithm, and would therefore help health care providers make informed treatment decisions [7,19,29,50]. Currently health care providers are advised to administer antimicrobials to patient with “an identified bacterial cause of fever” [34], which relies on the subjective assessment of the clinician without any diagnostic guidance. The addition of a specific assay, meeting the TPP specifications described here, would further allow the classification of patients as either “requiring” or “not requiring” antibiotics and reduce the excessive use of antibiotics.

In summary, the described TPP will guide the development of innovative diagnostic platforms that can help to distinguish between bacterial and non-bacterial infections, and guide individualized treatment decisions in LMICs. Although development of a suitable test is the first step, the working group is aware that large scale clinical trials with different populations and pre-test probability of bacterial infection are necessary to understand the final diagnostic performance in the target settings. However, with more than 90 million patients presenting to African health facilities with non-malarial fevers every year [4] the global health need is large and implementation of a tool could have large impact. The experts convened for this project agreed with previous analyses that a validated bacterial vs. non-bacterial assay is just the beginning, and TPPs for simple, rapid, pathogen-specific assays, especially for pathogens requiring specific antimicrobial choices (e.g., Gram-positive infections, pathogens resistant to standard therapy, tetracycline-responsive pathogens) as well as triage tests (e.g. need for referral of sever viral and/or bacterial infections) are also needed [19,50,51].

In line with Derda and colleagues we would like to reiterate the call for cross-continental collaborations and encourage all stakeholders (industry, academia, non-for-profit, non-governmental-organizations) to initiate collaborations early in the developmental, evaluation and validation process to allow for successful and timely technology advances of assays needed to save lives and reduce the threat of antimicrobial resistance [52].

## Supporting Information

S1 File 1^st^ TPP draft. (PDF)

S2 File Literature search details. (PDF)

S3 File 2^nd^ TPP draft. (PDF)

S4 File 3^rd^ TPP draft. (PDF)
