# Supplementary material for: Target Product Profile for a Diagnostic Assay to Differentiate between Bacterial and Non-Bacterial Infections and Reduce Antimicrobial Overuse in Resource-Limited Settings: An Expert Consensus
Source: PLoS One. 2016 Aug 25;11(8):e0161721. doi: 10.1371/journal.pone.0161721 (PMC4999186; doi:10.1371/journal.pone.0161721)
Supplement: S1 File — (PDF) [file pone.0161721.s001.pdf]

Supplementary 1: First draft TPP prepared by SD and shared prior to the biomarker meeting in September 2016

Population

**Minimal requirements:**<br>Adult (none-malarial) population with fever<br>OR<br>Children (none-malarial) population with fever	It is expected that patients that tested negative for with a malaria RDT would subsequently be tested with a biomarker test to determine if the infection has a viral or bacterial cause.<br>As biomarker cut-offs might be different for adults or children separate tests might be needed for the adult and children febrile population.
**Optimal requirements:**<br>Total febrile population	Ideally one device could be used to test all patients in a community health center without having to distinguish by age.

User

**Minimal requirements:**<br>Village health volunteer with minimal education and limited laboratory and medical training	It is expected that the device would be deployed at the lowest level of the health care system. At this level the staff has likely had very limited education without specific medical training.
**Optimal requirements:**<br>Trained lay person without medical training	

Format

**Minimal requirements:**<br>Biomarker testing alone	Rapid diagnostic tests build as lateral flow cassettes have been used for malaria diagnostic since the mid-1990. Health workers at community level are familiar with using such a device and a similar design for biomarker testing either integrated into a malaria RDT or as part of a diagnostic algorithm (malaria=neg -> biomarker).
**Optimal requirements:**<br>Biomarker integrated with malaria RDT	

Sample type/collection

**Minimal requirements:**<br>Whole blood from finger prick collected with a lancet	Lancet selection should consider trade-offs between cost, safety, user preference, and blood volume requirements. Biomarkers might be also found in saliva or buccal swabs (8), which represent less invasive samples, and the possibility of such should be explored, particularly for community use.
**Optimal requirements:**<br>Less invasive samples like saliva or buccal not requiring finger pricking	

Sample volume/sample transfer device

**Minimal requirements:**<br>1-100µL<br>Transfer device included in kit	A number of different transfer devices for finger prick blood have been developed and evaluated (9) for malaria RDTs.
**Optimal requirements:**<br>1-50µL<br>Transfer device included in kit	

Additional sample preparation

**Minimal requirements:**<br>1-2 additional steps	Sample preparation prior to applying the sample to the biomarker test should be kept to a minimum.<br>This will reduce the likelihood of error and will ensure higher reproducibility of results.
**Optimal requirements:**<br>None required	

Detection/Read-out

**Minimal requirements:**<br>Simple visual read-out (qualitative); clear result with a high-contrast band easily readable inside and outside; Alone or in conjunction with a simple battery powered reader (only if necessary)	Simple visual read-outs, which don't require additional interpretation, will be most suited for deployment at the community level.<br>Reading of the tests should ideally be possible without extra equipment, however an additional reader might be useful for record keeping and could be included with the device.
**Optimal requirements:**<br>Simple visual read-out; clear result with a high-contrast band easily readable inside and outside;	

Internal quality control

**Minimal requirements:**<br>Easily visible process control line;	Internal quality control to rule out false negative testing needs to be included for quality assurance reasons. After every valid test a control line appears to ensure the test
**Optimal requirements:**<br>Same	

	has been correctly performed. Only if the control line is visible the test can be reported as positive or negative.

Supplies needed

**Minimal requirements:**<br>No additional supplies needed.<br>All supplies are provided in self-contained kit	All reagents and tools needed to perform the biomarker test should be included in the provided in the kit.<br><br>Example from malaria RDTs which include the test cassette in a sealed sachet and buffer.
**Optimal requirements:**<br>Same	

Storage conditions

**Minimal requirements:**<br>Room temperature up to 50˚C	Lowest tier health centers will not have air conditioned storage facilities available, therefore test kits need to be stable at varying temperatures without refrigeration.
**Optimal requirements:**<br>Same	

Portability

**Minimal requirements:**<br>Highly portable	A test performed at community level needs to be highly portable to be performed near the bedside or near the patients (field, house) without having to transport samples to a central facility.
**Optimal requirements:**<br>Same	

Bio-Safety

**Minimal requirements:**<br>Closed format to be disposed as infectious waste<br><br>In case viral hemorrhagic fevers are a possible causes of a viral disease, specific standard operating procedures need to be in place to protect health care staff and to allow fast public health intervention.	Patients are potentially infected with contagious pathogens therefor all waste generated in the process of testing needs to be disposed of as infectious waste.<br>The assay should be closed so that minimal handling of the sample and test is required to ensure safety for the health worker and the environment.
**Optimal requirements:**<br>Same	

Analytical sensitivity / Limit of detection (LoD)

**Minimal requirements:**<br>To be determined, depending on the biomarker in question.	The analytical sensitivity describes the ability of the test to detect small quantities of the biomarker/molecule in question.
**Optimal requirements:**<br>See above.d	

Diagnostic sensitivity

**Minimal requirements:**<br>80%	The diagnostic sensitivity will very much depend on the target population, background levels of disease and the day of presentation at the health facility.
**Optimal requirements:**<br>99%	

Diagnostic specificity

**Minimal requirements:**<br>90%	To obtain high specificity it is a trade-off with sensitivity. Similar to the sensitivity, the specificity will likely be depended on the population, background illness and the day of illness the patient presents to the health facility.
**Optimal requirements:**<br>99%	

Time to result

**Minimal requirements:**<br>Maximum 30 minutes	At community level the turn-around time needs to be quick to<br>allow enough time to triage patients to a higher tier health facility without risking health consequences.
**Optimal requirements:**<br>Less than 15 minutes	

Throughput

**Minimal requirements:**<br>Individual testing (1 test / patient)<br>~ 8 tests per hour similar to malaria RDT	Devices used at community level to guide patient management need to packed individually and be used for individual patients.

**Optimal requirements:**<br>Individual testing (1 test / patient)<br>More than 10 tests per hour	For large scale screening studies batching samples would be beneficial.

Target shelf life

**Minimal requirements:**<br>18 months at temperatures between 2°C and 30°C; stable for 2 weeks at 40°C.	Stock control and the expiration of reagents is a major problem in resource-poor laboratories therefor a biomarker assay needs to have a reasonable long shelf life to allow stock piling on site and centrally.
**Optimal requirements:**<br>36 months at temperatures between 2°C and 40°C; stable for 2 weeks at 50°C; time-temperature monitors included on each kit.	

Ease of use

**Minimal requirements:**<br>Not more than 2 timed steps during assay performance;<br>Instructions should include a diagram of the method and result interpretation.	As tests are aimed at staff or lay personal with limited training the number of steps to perform the test need to be kept to a minimum.<br>This will reduce the error rate and increase reproducibility of results.
**Optimal requirements:**<br>One or no timed step during the assay.<br>Instructions should include a diagram of the method and result interpretation.	

Interpretation

**Minimal requirements:**<br>Clear positive / negative read-out clearly indicated in the instructions. No room for interpretation error.	Tests are aimed at staff with limited training and therefor the interpretation needs to be clear without any room for interpretation. This will reduce error rates and avoid false results and misdiagnosis and the wrong treatment.
**Optimal requirements:**<br>Same.	

Operation temperature

**Minimal requirements:**<br>20-35˚C (up to 90% humidity)	No specialized facilities with air-conditioning are available at community level.<br>Tests need to withstand the conditions in the field without quality loss.
**Optimal requirements:**<br>10-40˚C (up to 90% humidity)	

Data handling

**Minimal requirements:**<br>None.	At community level no special data management capability is needed. Recording should be done for surveillance purposes but this does not be integrated in the device.
**Optimal requirements:**<br>Same	

Shipping conditions

**Minimal requirements:**<br>Conformance to applicable requirements of ASTM D4169-05 and ISO 11607-1: 2006.	Shipping validation is mandatory for medical devices that are CE marked.
**Optimal requirements:**<br>Same	

Training requirements for user

**Minimal requirements:**<br>Less than one day of training for VHV or lay person	Training requirements need to be limited and similar to the requirements for malaria RDTs.
**Optimal requirements:**<br>One hour or less training required for VHV or lay person	

Instrumentation requirements

**Minimal requirements:**<br>No reader needed.	No additional instruments should be required at the community level. Any further device might reduce the usability of a test in the field.
**Optimal requirements:**<br>Same	

Training requirements for user

**Minimal requirements:**<br>1 day or less training for VHV or lay person	Joint training of medical and laboratory staff should be undertaken to ensure the translation of laboratory biomarker results in treatment changes.
**Optimal requirements:**	

1 hour of training for VHV or lay person	

Instrumentation requirements

**Minimal requirements:**<br>Not applicable.	A simple rapid test should be used, not requiring any instruments.
**Optimal requirements:**<br>Same	

Power requirement

**Minimal requirements:**<br>No power required	As stable power supply cannot be expected at community level the device needs to be independent of electricity.
**Optimal requirements:**<br>Same	

Water requirement

**Minimal requirements:**<br>No water required	All buffers and solutions need to be included in the device as continuing water supply cannot be expected at this level of the health care system.
**Optimal requirements:**<br>Same	

Cost

**Minimal requirements:**<br>Similar to malaria RDT (~2 USD)<br>Same or cheaper than a full course of antibiotics	A diagnostic tool to identify biomarkers indicative of viral or bacterial infections needs to be priced similar to a malaria RDT or the cost of antibiotic treatment course to be used in the field.
**Optimal requirements:**<br>Less than 1 USD	
