# Supplementary material for: Target Product Profile for a Diagnostic Assay to Differentiate between Bacterial and Non-Bacterial Infections and Reduce Antimicrobial Overuse in Resource-Limited Settings: An Expert Consensus
Source: PLoS One. 2016 Aug 25;11(8):e0161721. doi: 10.1371/journal.pone.0161721 (PMC4999186; doi:10.1371/journal.pone.0161721)
Supplement: S2 File — (PDF) [file pone.0161721.s002.pdf]

Supplementary table 2: Details of the literature search terms (where applicable) and the chosen reference. Reasoning for deciding on certain characteristics are given in the text. "Not applicable" indicates that context specific parameters were needed that were proposed by SD and discussed and agreed by all authors.

**Characteristic**	**Search terms**	**Chosen reference**
Target population	fever AND/OR population AND/OR incidence AND/OR epidemiology AND/OR cases	Gething *et. al.* 2010; WHO 2014 (IMCI);<br>Feikin *et. al.* 2011
Target level of health system	Not applicable.	Maputo Declaration 2008
Target user	Clinical laboratory AND/OR Africa AND/OR low and middle income countries AND/OR microbiology AND/OR resource limited AND/OR diagnostic laboratory	Petti *et. al.* 2006
Price of one test	Not applicable.	Expert consensus
Diagnostic sensitivity	Sensitivity, specificity AND/OR diagnostic AND/OR impact	Lim *et. al.* 2006<br>Expert consensus
Diagnostic specificity		
Multiplexing	Not applicable.	Expert consensus
Ease of test performance	Not applicable.	Expert consensus
Sample type	Not applicable.	Expert consensus
Volume	Not applicable.	Expert consensus
Sample collection	Not applicable.	Industry standards
Additional sample preparation	Not applicable.	Expert consensus
Kit configuration	Not applicable.	Industry standards
Batch/Quality control	Not applicable.	Expert consensus
Process control	Not applicable.	Expert consensus
Reagent preparation	Target product profile AND/OR reagents AND/OR steps	Chua *et. al.* 2015
Time to result (per sample)	Not applicable	Expert consensus
Hands on time	Not applicable.	Expert consensus
Sample throughput	Not applicable.	Expert consensus
Result stability	Not applicable.	Industry standard
Assay type	Not applicable.	Expert consensus
Biosafety and waste disposal	Not applicable.	Laboratory Biosafety Manual, WHO 2014
Storage conditions	Target product profile AND/OR storage AND/OR tropical temperatures AND/OR hot AND/OR low and middle income countries	Chua *et. al*. 2015
Operation conditions	Target product profile AND/OR operation AND/OR tropical temperatures AND/OR hot AND/OR low and middle income countries	Chua *et. al*. 2015
Shipping conditions	Target product profile AND/OR operation AND/OR tropical temperatures AND/OR hot AND/OR low and middle income countries	Expert consensus

Training requirements	Target product profile AND/OR training AND/OR point of care AND/OR diagnostic AND/OR low and middle income countries	Chua *et. al.* 2015
Equipment	Not applicable.	Expert consensus
Power supply	Target product profile AND/OR power AND/OR point of care AND/OR diagnostic AND/OR low and middle income countries	Chua *et. al.* 2015
Water supply	Not applicable	Industry standards
External maintenance	Not applicable	Industry standards
Calibration	Not applicable	Industry standards
Data interpretation/ output	Not applicable	Expert consensus
Connectivity	FIND internal documentation	Isaak *et. al.* in preperation
