# Supplementary material for: Target Product Profile for a Diagnostic Assay to Differentiate between Bacterial and Non-Bacterial Infections and Reduce Antimicrobial Overuse in Resource-Limited Settings: An Expert Consensus
Source: PLoS One. 2016 Aug 25;11(8):e0161721. doi: 10.1371/journal.pone.0161721 (PMC4999186; doi:10.1371/journal.pone.0161721)
Supplement: S3 File — (PDF) [file pone.0161721.s003.pdf]

Supplementary 3: Second draft TPP (November 2015) prepared by SD and shared with the working group after the biomarker meeting.

Goal

Rapid, biomarker-best testing to differentiate between viral and bacterial infections.	**Explanation:**<br>A simple test that is easily accessible and can be used at community level outside of centralized health facilities. The aim is to triage patients to withhold antimicrobial therapy from patients with likely viral infections. In contrast patients with likely bacterial infections can be referred to higher tier facilities for further testing or be treated using antibiotics appropriate for the local epidemiology.

Target population

**Minimal requirements:**<br>Depending on the biomarker in question, adult or children with non-severe, non-malarial fever presenting at community level.	The targeted population will present to outpatient clinics, health centers or lower tier health facilities (e.g. district level).<br>The ease of use and characteristics of the biomarker test will determine how broad a test is applicable.
**Optimal requirements:**<br>Total febrile population presenting at community level with suspected non-malarial fever.	

Target user of test

**Minimal requirements:**<br>Health workers with laboratory and medical training	Under the ideal circumstances a biomarker-test can be used at village outpatient level and at this level the health providers have likely had very limited education without specific medical training in biomarker diagnostic.
**Optimal requirements:**<br>Trained lay person without medical training	

Price of individual testing

**Minimal requirements:**<br><10USD	No good evidence based data are available to estimate a minimal price requirement. However, given that it would replace repeated individual pathogen testing a minimum price of ~2x the average dengue RDT (23) is assumed as the minimum price requirement.<br><br>Under ideal circumstances a simple biomarker-test is deployable in a variety of geographical settings. The resulting large market would contribute to reduced production costs.
**Optimal requirements:**<br>Similar to malaria RDT (<1 USD)	

Analytical sensitivity / Limit of detection (LoD)

**Minimal requirements:**<br>Depending on biomarker tested.	The analytical sensitivity describes the ability of the test to detect small quantities of the biomarker/molecule in question. This parameter will largely depend on the chosen biomarker and has to be determined for each biomarker of interest in order to determine correct cut-off points.
**Optimal requirements:**<br>Depending on biomarker tested.	

Diagnostic sensitivity to differentiate bacterial and viral infections

**Minimal requirements:**<br>Equal or better than 90%	The diagnostic sensitivity will very much depend on the target population, background levels of disease/biomarker and the day of presentation at the health facility.<br>What is an acceptable sensitivity to detect a bacterial infections is very much depended on what is considered an acceptable 'false negativity', which would result in under treatment or severe disease outcomes and death.<br>With a test that would be 90% sensitivity to correctly identify a bacterial infection 100 false negatives would be considered acceptable in a population of 1000 patients.
**Optimal requirements:**<br>Equal or better than 98%	

Diagnostic specificity

**Minimal requirements:**<br>Equal or better than 90%	Similar to the sensitivity, the specificity will likely be depended on the population, background illness and the day of illness the patient presents to the health facility.
**Optimal requirements:**<br>Equal or better than 99%	

	It the case of a biomarker test that aims to differentiate between non-bacterial and bacterial infections, limited specificity would refer to the misclassification of a non-bacterial as a bacterial infection, or vice versa.

Quantitation

**Minimal requirements:**<br>Qualitative	The read-out of a simple biomarker test should be simple without the need for further analysis of quantitative data.
**Optimal requirements:**<br>Qualitative	

Sample type/collection

**Minimal requirements:**<br>Whole blood from finger prick collected with a lancet	Lancet selection should consider trade-offs between cost, safety, user preference, and blood volume requirements.<br><br>Biomarkers might be also found in saliva or buccal swabs (24), which represent less invasive samples, and the possibility of such should be explored, particularly for community use.
**Optimal requirements:**<br>Less invasive samples like saliva or buccal not requiring finger pricking	

Sample volume/sample transfer device

**Minimal requirements:**<br>10–75µL of finger prick blood.<br><br>~0.2-1mL for saliva.<br>Transfer device included in kit	The sample volume needed for the test should be as small as possible to accommodate possible small blood or sputum volumes obtained from individual subjects.<br>A number of different transfer devices for finger prick blood have been developed and evaluated (25) for malaria RDTs.
**Optimal requirements:**<br>5-50µL of finger prick blood.<br>~0.2-1mL for saliva.<br>Transfer device included in kit	

Additional sample preparation

**Minimal requirements:**<br>1-2 sample-processing steps	Sample preparation prior to applying the sample to the biomarker test should be kept to a minimum, as this will reduce the likelihood of error and will ensure higher reproducibility of results.<br><br>Further given that limited laboratory facilities as well as human capacity will be available at community level any additionally required equipment will make the test more expensive and less likely to be performed at small health centers.
**Optimal requirements:**<br>None required	

Detection

**Minimal requirements:**<br>Alone or in conjunction with a simple battery powered reader	Simple visual read-outs, which don't require additional interpretation, will be most suited for deployment at the community level.<br>Reading of the tests should ideally be possible without extra equipment, however an additional reader could be included with the device.
**Optimal requirements:**<br>No additional reader or equipment necessary	

Additional reagents needed

**Minimal requirements:**<br>No additional supplies or reagents are needed. All supplies are provided in self-contained kit	All reagents and tools needed to perform the biomarker test should be included in the provided in the kit.<br>Example from malaria RDTs which include the test cassette in a sealed sachet and buffer.
**Optimal requirements:**<br>Same	

Time to result

**Minimal requirements:**<br>Less than 1h with less hands-on time	At community level the turn-around time needs to be quick to allow the result and patient management within the same visit.
**Optimal requirements:**<br>Less than 20 minutes	

Sample capacity

**Minimal requirements:**<br>One sample at a time	Ideally it should be possible to perform multiple tests at the same time to allow for patient influx due to seasonality.
Optimal requirements:<br>One and multiple sample at a time	

Bio-Safety

**Minimal requirements:**<br>No need for a biosafety cabinet; direct disposal of consumables	Biosafety cabinets and other safety equipment will not be available at low tier health facilities; hence in order to allow widespread use of the test no specific safety equipment should be needed.
**Optimal requirements:**<br>No need for a biosafety cabinet; direct disposal of consumables	

Waste disposal (solid)

**Minimal requirements:**<br>Simple trash; recyclable or compostable plastics/consumables	Simple waste disposal will allow the use of the test in peripheral facilities without infectious waste disposal capacity.
**Optimal requirements:**<br>Simple trash; recyclable or compostable plastics/consumables	

Waste disposal (infectious)

**Minimal requirements:**<br>Incineration or autoclaving of infectious material	Simple waste disposal will allow the use of the test in peripheral facilities without infectious waste disposal capacity.
**Optimal requirements:**<br>Not necessary	

Multi-use platform

**Minimal requirements:**<br>Biomarker test alone	Malaria detection remains important despite the decline of the infection, hence a biomarker test that is combined with malaria testing might be beneficial in certain settings. Further, a number of bacterial infections require specialized treatment as the organisms are not susceptible to most empiric treatments (e.g. *O. tsutsugamushi*, *Rickettsia* spp., *C. burnettii*).
**Optimal requirements:**<br>Biomarker testing in combination with specific detection of pathogens of local importance and/or pathogens that would require specialized treatment.	

Ease of test performance

**Minimal requirements:**<br>Not more than 2 timed steps during assay performance;<br>Instructions should include a diagram of the method and result interpretation.	As tests are aimed at staff or lay personal with limited training the number of steps to perform the test need to be kept to a minimum.<br>This will reduce the error rate and increase reproducibility of results.
**Optimal requirements:**<br>One or no timed step during the assay.<br>Instructions should include a diagram of the method and result interpretation.	

Storage conditions

**Minimal requirements:**<br>18 months at temperatures between 5°C and 35°C; no cold chain required	Stock control and the expiration of reagents is a major problem in resource-poor laboratories therefor a biomarker assay needs to have a reasonable long shelf life to allow stock piling on site and centrally.
**Optimal requirements:**<br>36 months at temperatures between 5°C and 45°C; stable for 2 weeks at 50°C; time-temperature monitors included on each kit; no cold chain required	

Operating temperature

**Minimal requirements:**<br>Between 15°C and 40°C; up to 70% humidity	No specialized facilities with air-conditioning are available at community level.<br>Tests need to withstand the temperature fluctuations in the field without quality loss.
**Optimal requirements:**<br>Between 15°C and 45°C ; up to 90% humidity	

Shipping conditions

**Minimal requirements:**	

All included in the kit (including water, alcohol, lancet, swab, test); shipping without cold chain	It is important that the biomarker test can be shipped without the need for cold transport (dry ice).
**Optimal requirements:**<br>All included in the kit (including water, alcohol, lancet, swab, test); shipping without cold chain	

Training & education needs

**Minimal requirements:**<br><3 days, healthcare worker	Low training and education needs are necessary given high staff turn-around.
**Optimal requirements:**<br><1 day, healthcare worker	

Instrumentation requirements

**Minimal requirements:**<br>Single tool used alone or in conjunction with a reader	Only minimal additional instruments should be required at the community level. Any further device might reduce the usability of a test in the field.
**Optimal requirements:**<br>Preferably instrument free.<br>If instrument: Small, portable or hand-held instrument (<1kg) that can operate on battery or solar in places with interrupted power supply	

Power requirement

**Minimal requirements:**<br>Optional battery or solar operation	As stable power supply cannot be expected at community level the device needs to be independent of the grid.<br>The less infrastructural requirements are needed the more widely applicable will the biomarker test be.
**Optimal requirements:**<br>None	

Water requirement

**Minimal requirements:**<br>No water required	All buffers and solutions need to be included in the device as continuing water supply cannot be expected at community level.
**Optimal requirements:**<br>No water required	

Maintenance (external)

**Minimal requirements:**<br>Preventative maintenance at 1 year or >1000 samples; simple with only minimal expertise; Maintenance alert should be included	Maintenance of instruments is important and necessary, however in resource poor settings regular technical is rarely available.<br>A timely alert for the user will help to keep within maintenance schedules. Such an alert is particularly important if the device is not always used by the same person and accurate record keeping is unlikely.
**Optimal requirements:**<br>Disposal, no maintenance required	

Instrumentation requirements

**Minimal requirements:**<br>Single tool used alone or in conjunction with a reader	Only minimal additional instruments should be required at the community level. Any further device might reduce the usability of a test in the field.
**Optimal requirements:**<br>Preferably instrument free.<br>If instrument: Small, portable or hand-held instrument (<1kg) that can operate on battery or solar in places with interrupted power supply	

Power requirement

**Minimal requirements:**<br>Optional battery or solar operation	As stable power supply cannot be expected at community level the device needs to be independent of the grid.<br>The less infrastructural requirements are needed the more widely applicable will the biomarker test be.
**Optimal requirements:**<br>None	

Water requirement

**Minimal requirements:**<br>No water required	All buffers and solutions need to be included in the device as continuing water supply cannot be expected at community level.
**Optimal requirements:**<br>No water required	

Maintenance (external)

**Minimal requirements:**	

Preventative maintenance at 1 year or >1000 samples; simple with only minimal expertise; Maintenance alert should be included<br>**Optimal requirements:**<br>Disposal, no maintenance required	Maintenance of instruments is important and necessary, however in resource poor settings regular technical is rarely available.<br>A timely alert for the user will help to keep within maintenance schedules. Such an alert is particularly important if the device is not always used by the same person and accurate record keeping is unlikely.

Calibration

**Minimal requirements:**<br>Remote calibration or auto-calibration<br>**Optimal requirements:**<br>None required	Onsite calibration should not be required as it leaves additional room for error which might subsequently result in misinterpretation of biomarker results.

Quality control/Internal controls

**Minimal requirements:**<br>Easily visible process control<br>**Optimal requirements:**<br>Easily visible process and performance control	Internal quality control to rule out false negative testing needs to be included for quality assurance reasons.<br>After every valid test a control line appears to ensure the test has been correctly performed. Only if the control line is visible the test can be reported as positive or negative.<br>In addition to internal controls, EQA is important.

Data display/Result capturing

**Minimal requirements:**<br>In case instrumentation is needed to read the results, the instrument should have a simple LCD screen, key pad or touch screen with a display that visually guides the user (accommodating limited literacy)<br><br>Ability to save results on instrument/reader or remotely.<br>**Optimal requirements:**<br>Simple read-out without the need for additional instrumentation. Manual result capturing.	At community level any interaction with a device or data handling needs to be as simple as possible to account for limited language skills.

Portability

**Minimal requirements:**<br>Semi-portable or stationary at a dedicated place within the facility<br>**Optimal requirements:**<br>Highly Portable	Ideally a test performed at community level would be portable to move the device near the patients (field, house) without having to transport samples to a central facility.

Regulatory requirements

**Minimal requirements:**<br>Assay and system manufactured in compliance with ISO EN 13485 or higher standards and/or regulations and in compliance with ISO IEC 62304 Medical Device Data Systems.<br>Manufacturing facility certified and authorized for use by a regulatory authority that is a member of the International Medical Device Regulators Forum (IMDRF) formerly known as Global Harmonization Task Force (GHTF); registered for in vitro diagnostic use.<br>**Optimal requirements:**<br>Assay and system manufactured in compliance with ISO EN 13485 or higher standards and/or regulations and in compliance with ISO IEC 62304 Medical Device Data Systems.<br>Manufacturing facility certified and authorized for use by a regulatory authority that is a member of the International Medical Device Regulators Forum (IMDRF) formerly known as Global	

Harmonization Task Force (GHTF); registered for in vitro diagnostic use.	

Data export in case instrumentation is required

**Minimal requirements:**<br>Full data export (on usage of device, errors, protected results data) over USB port and network. Network connectivity through Ethernet, Wi-Fi, and/or GSM/UMTS mobile broadband modem. Results should be encoded using a documented standard (such as HL7) and be formatted as JSON text. JSON data should be transmitted through HTTP(S) to a local or remote server as results are generated. Results should be locally stored and queued during network interruptions and sent as a batch when connectivity is restored.	Data export will allow the integration of the test into national surveillance networks. It will further allow for supply chain management.
**Optimal requirements:**<br>Preferably the biomarker test would be instrument free.<br>However, data export might still be possible, for instance via mobile phone reader.<br><br>Full data export (on usage of device, errors, protected results data) over USB port and network. Network connectivity through Ethernet, Wi-Fi, and/or GSM/UMTS mobile broadband modem. Results should be encoded using a documented standard (such as HL7) and be formatted as JSON text. JSON data should be transmitted through HTTP(S) to a local or remote server as results are generated. Results should be locally stored and queued during network interruptions and sent as a batch when connectivity is restored.	

Data analysis

**Minimal requirements:**<br>Manual data analysis in case of a instrument-free assay.<br>Integrated in case of an instrument-based assay.	Integrated data analysis particularly in automated systems will reduce interpretation error.
**Optimal requirements:**<br>Manual data analysis in case of a instrument-free assay.<br>Integrated in case of an instrument-based assay.	

Electronics and software

**Minimal requirements:**<br>Integrated	
**Optimal requirements:**<br>None needed	
