# Supplementary material for: Target Product Profile for a Diagnostic Assay to Differentiate between Bacterial and Non-Bacterial Infections and Reduce Antimicrobial Overuse in Resource-Limited Settings: An Expert Consensus
Source: PLoS One. 2016 Aug 25;11(8):e0161721. doi: 10.1371/journal.pone.0161721 (PMC4999186; doi:10.1371/journal.pone.0161721)
Supplement: S4 File — (PDF) [file pone.0161721.s004.pdf]

Supplementary 4: Third draft TPP (January 2016) prepared by SD and shared with the working group prior to the face-to-face meeting.

Characteristic	Acceptable ("must")	Desired ("want")	Reference
**SCOPE**			
**Goal**	Rapid, biomarker-based best testing to differentiate between bacterial and non-bacterial infections to guide antimicrobial treatment.		Expert consensus
**Target population**	Children with non-severe, non-malarial fever presenting at (what?)	Total febrile population presenting with fever.	Gething *et. al.* 2010; WHO 2014 (IMCI);
**Target level of health system**	Tier 1 low level (passive case finding)		Maputo Declaration 2008
**Target user**	Healthcare worker	Trained lay person	Petti *et. al.* 2006
**Price of individual test**	10 USD	< 2 USD or equivalent to a course of antibiotic treatment	Expert consensus
**Analytical sensitivity/LoD**	Detection of a relevant bacterial/viral infection during the acute phase of the illness.	Detection of a relevant bacterial infection alone or as a co-infection with malaria, during the acute phase of the illness.	Not applicable
**Diagnostic sensitivity to differentiate between bacterial and non-bacterial infections**	Equal or better than 95%	Equal or better than 99%	Expert consensus
**Diagnostic specificity**	Equal or better than 80%	Equal or better than 90%	Expert consensus
**TEST PROCEDURE**			
**Multiplexing**	Marker(s)	Marker(s) plus pathogen specific testing (e.g. malaria)	Expert consensus
**Ease of test performance**	Not more than 2 timed steps during assay performance	One or no timed step during the assay.	Expert consensus
**Sample type**	Capillary blood<br>Saliva		Expert consensus
**Volume**	100µL (~ 4 drops) for older children and adults; 50µL (~2 drops) for young children of capillary blood<br>~0.2-1mL for saliva.	25µL (~1 drop) of capillary blood<br>~0.2-1mL for saliva.	Expert consensus
**Collection**	Transfer device included in the test		Community based consensus
**Additional sample preparation**	1 sample-processing steps	None required	Expert consensus
**Kit configuration**	No additional reagents outside of the kit required		Community based consensus

**Control material**	Positive and negative and internal control provided with each test.		Community based consensus
**Reagent preparation**	Minimal of one additional step to prepare prior to use<br>No (precise) measuring required.	No additional reagents required, everything is provided ready-to-use	Chua *et. al.* 2015
**Time to result (per sample)**	Less than 60min	Less than 20min	WHO meeting report-TPP for TB diagnostic
**Hands on time**	Total hands-on-time should be less than 5 min	Total hands on time should be less than 3 min	Expert consensus
**Sample capacity**	Capacity for parallel processing of <10 samples (?)		Expert consensus
**Result stability**	30 min	1 hour	n.a.
**Analysis type**	Qualitative	Quantitative	Expert consensus
**OPERATIONAL CHARACTERISTICS**			
**Biosafety**	No need for a biosafety cabinet; basic safety procedures need to be followed (standard PPE)		Laboratory Biosafety Manual, WHO 2014
**Waste disposal**	Biohazard waste	Testing device or compostable plastics for minimal environmental impact	n.a.
**Storage conditions**	12 month at flactuating temperature (5-40°C)<br>Up to 90% relative humidity<br>No controlled temperature required	24 month at flactuating temperature (5-50°C)<br>Up to 90% relative humidity<br>No controlled temperature required	Chua *et. al.* 2015
**Operation conditions**	Between 5°C - 40°C<br>Up to 90% humidity	Between 5°C - 50°C<br>Up to 90% humidity	Chua *et. al.* 2015
**Shipping conditions**	Shipping without cold chain		Expert consensus
**Training requirements**	Less than 2 days including proficiency panel	Less than half a day including proficiency panel	Chua *et. al.* 2015
**Equipment (instrument external to test)**	Small, portable or hand-held integrated instrument (<1kg) that must operate on battery	Instrumentation free.	Expert consensus
**Power supply**	Battery or solar powered	None required.	Chua *et. al.* 2015
**Water supply**	No external water required.		Community based consensus
**External maintenance**	Preventative maintenance at 2 year or >1000 samples; simple with only minimal expertise<br>Maintenance alert should be included.	None required	Community based consensus
**Calibration**	Remote calibration or auto-calibration	No calibration required	Community based consensus
**Internal process controls**	Included		Chua *et. al.* 2015
**Data interpretation and output**	No interpretation required by user		Expert consensus

**Connectivity**	Wireless connectivity required	TPP in development at FIND/WHO
